# Cardiovascular Disease Mortality Risk among Long-term Survivors of Different Hodgkin Lymphoma Types

**DOI:** 10.31083/RCM24981

**Published:** 2025-06-30

**Authors:** Zhenxing Lu, Liyu Guo, Huijuan He, Linglong Liu, Manting Feng, Xueqi Xiao, Xin Lin, Yingyu Deng, Tianwang Guan, Xiaoping Peng

**Affiliations:** ^1^Department of Cardiology, The First Affiliated Hospital, Jiangxi Medical College, Nanchang University, 330006 Nanchang, Jiangxi, China; ^2^The Second Clinical Medical College, Southern Medical University, 510280 Guangzhou, Guangdong, China; ^3^Cardio-Oncology Group, Medical Exploration and Translation Team, 510000 Guangzhou, Guangdong, China; ^4^Department of Anesthesiology, The Second Clinical College of Guangzhou Medical University, 510182 Guangzhou, Guangdong, China; ^5^Department of Clinical Medicine, The Second Clinical College of Guangzhou Medical University, 510182 Guangzhou, Guangdong, China; ^6^Department of Radiation Oncology, Cancer Center, The Tenth Affiliated Hospital, Southern Medical University (Dongguan People’s Hospital), 523059 Dongguan, Guangdong, China

**Keywords:** cardiovascular disease, Hodgkin lymphoma, subtypes, mortality, temporal trend

## Abstract

**Background:**

The temporal trend and disparities in cardiovascular disease (CVD) mortality risk among long-term survivors of different Hodgkin lymphoma (HL) types are unclear. Therefore, we aimed to examine the temporal trend and disparities in CVD mortality risk among survivors of various HL subtypes.

**Methods:**

This multicenter cohort included 20,423 patients with HL diagnosed between 1975 and 2018, with an average follow-up time of 18.5 years. Proportional mortality ratio, cumulative cause-specific mortality accounting for competing risks, standardized mortality ratio, and absolute excess risk were calculated.

**Results:**

Patients with nodular lymphocyte-predominant HL (NLPHL) and classical HL exhibited higher CVD-related deaths than HL-related deaths after approximately 12 and 120 months of follow-up, respectively. From the initial diagnosis to >500 months of follow-up, the cumulative CVD mortality increased continuously without a plateau and exceeded that of HL at different times in most patients with various HL types. However, CVD mortality risk exceeded that of HL earlier in NLPHL than in other types. Black or male patients with nodular sclerosing classical HL exhibited a higher CVD mortality risk, while a contrary trend was noted among those with lymphocyte-rich classical HL or lymphocyte-depleted classical HL. Over the past decades, CVD mortality risk has decreased slowly or remained unchanged. Patients with HL exhibited higher risks of CVD mortality than the general population.

**Conclusions:**

CVD mortality risk exceeded that of HL over time among many survivors. This temporal trend was significantly different among various HL subtypes. Thus, more effective strategies are required to reduce the risk of CVD mortality, depending on subtypes.

## 1. Introduction

Hodgkin lymphoma (HL) was initially described by the famous British pathologist Thomas Hodgkin in 1832. HL accounts for approximately 0.6% of all cancers and 10% of lymphomas worldwide [1, 2, 3]. HL is divided into classical and nodular lymphocyte-predominant HL (NLPHL) types based on morphology and immunohistochemistry. Classical HL is further subdivided into nodular sclerosing classical HL (NSCHL), mixed-cellularity classical HL (MCCHL), lymphocyte-rich classical HL (LRCHL), and lymphocyte-depleted classical HL (LDCHL) subtypes. As treatment has improved over the past decades, the cure rate of HL has reached 80%–90% in some populations, meaning this malignancy has become highly treatable [4, 5]. However, successful HL treatment is associated with an increased risk of morbidity and mortality from other diseases among long-term survivors, including secondary neoplasms, cardiovascular disease (CVD), infections, and endocrine and respiratory disorders [6, 7, 8, 9, 10, 11].

Previous studies reported that patients with HL exhibited a high risk of treatment-related cardiovascular complications, including coronary artery disease, valvular dysfunction, diastolic dysfunction, congestive heart failure, and pericardial disease [12, 13, 14, 15]. Indeed, CVD has become one of the most important causes of death among long-term survivors of HL [7, 16, 17, 18, 19]. Previous studies that assessed the risk of cause-specific mortality for patients with HL categorized this as a single population. However, various HL subtypes may differ based on cause-specific mortality in the setting of similar clinical management. Moreover, few studies examined the temporal trend and disparities in cause-specific mortality risk among patients with different HL subtypes owing to the difficulty in assessing the CVD-specific mortality risk in various populations. Additionally, whether the risk of death due to CVD exceeds that due to secondary malignancy or the primary tumor based on the current markedly improved HL cure rate is unclear. HL incidence peaked first around 20–30 years and again around 50–70 years of age [20]. Older patients have a lower curative rate than younger patients. Thus, it is necessary to cover all ages to obtain a comprehensive assessment of the patients with HL. However, few studies have conducted this evaluation [21, 22, 23].

Therefore, we conducted this study among a large, multicenter cohort of 20,423 patients of all ages diagnosed with HL between 1975 and 2018 to examine the temporal trend and disparities in CVD mortality risk among survivors of various HL subtypes. This study can facilitate a more precise assessment of CVD mortality risk among these patients in the clinical setting and improve their long-term survival and quality of life.

## 2. Materials and Methods

### 2.1 Data Source

This study used data from the National Cancer Institute’s Surveillance, Epidemiology, and End Results (SEER) database (available at https://seer.cancer.gov/). The SEER program is a publicly available, authoritative database with quality-assured data because of its systematic data collection procedure [24]. The SEER database provides information on cancer statistics to reduce the cancer burden among the U.S. population. Data were extracted from the SEER 9 registry, covering most years. SEER*Stat software (version 8.4.1; National Cancer Institute, National Institutes of Health, Bethesda, MD, USA, available from https://seer.cancer.gov/seerstat/) was used to create a case list. Ethical approval for our study was not required, as only de-identified data were used.

### 2.2 Study Population

Patients with a definitive diagnosis of a single primary HL subtype who actively attended follow-up assessments and were first diagnosed between 1975 and 2018 were included in this study. When analyzing proportional mortality ratio (PMR), cumulative cause-specific disease, standardized mortality ratio (SMR), and absolute excess risk (AER), patients with follow-up times of less than two months or of unknown race were excluded from the analysis [25].

### 2.3 Patient Variables and Outcomes

This study considered various causes of death, including infection, diabetes mellitus, Alzheimer’s disease, respiratory disease, digestive disease, kidney disease, and other undefined non-neoplastic diseases and suicides, accidents, and homicide. CVD encompassed heart disease, cerebrovascular disease, atherosclerosis, hypertension without heart disease, aortic aneurysm and dissection, and other diseases of the arteries, arterioles, or capillaries, classified according to the International Classification of Disease-10 codes (**Supplementary Table 1**). Variables were classified as follows: age at diagnosis (0–34 years; 35–64 years; 65+ years) [19]; sex (male or female); race (white; black; other); year of diagnosis (1975–1988; 1989–2002; 2003–2018); follow-up intervals in months (2–11; 12–59; 60–119; 120–179; 180+); HL pathological subtypes (NSCHL; MCCHL; LRCHL; LDCHL; NLPHL).

Primary outcomes were death from HL, other neoplasms, CVD, or other non-neoplastic etiologies. The initial follow-up time was counted from the HL diagnosis date, and the follow-up termination time was upon all-cause death or definitive loss to follow-up.

### 2.4 Statistical Analyses

Data are represented using descriptive statistics according to age, sex, race, age at diagnosis, year of diagnosis, follow-up interval, and HL subtype. The PMR was defined as the number of deaths from any one cause (HL, CVD, other neoplasms, other non-neoplasms) divided by the total number of deaths [26]. SMR was defined as the ratio of observed deaths to the expected number of deaths, with the expected number of deaths obtained by multiplying the number of person-years at risk by the mortality rate of the general population with the same age, sex, race, and calendar period. The mortality rate for the general population was derived from the Centers for Disease Control and Prevention in the United States (https://wonder.cdc.gov/). AER was calculated by subtracting the expected deaths from the observed number divided by the number of person-years at risk with results multiplied by 10,000 and expressed as per 10,000 person-years. A corresponding 95% confidence interval (CI) was calculated for the SMR. All statistical analyses were performed using the IBM Statistical Package for the Social Sciences (version 26; IBM Corp., Chicago, IL, USA) and R (version 4.0.3; R Core Team, Vienna, Austria, 2020-10-10) software packages.

## 3. Results

### 3.1 Patient Characteristics

Our cohort included 20,423 patients with HL diagnosed between 1975 and 2018. Of the 20,423 patients with HL, 11,389 (55.8%) were male, and 9034 (44.2%) were female. White and black patients accounted for 85.5% and 10% of the population, respectively; others accounted for 4.5%. Patients aged 0–34, 35–64, and 65+ years were 57.7%, 31.8%, and 10.5%, respectively. The proportions of patients diagnosed during each time interval were 27.3% (1975–1988), 33.6% (1989–2002), and 39.1% (2003–2018). Among our studied population, NSCHL, MCCHL, LRCHL, LDCHL, and NLPHL subtype diagnoses accounted for 70.8%, 18.1%, 4.0%, 2.2%, and 4.9% of patients, respectively. Average follow-up time was 18.5 years (Table 1). **Supplementary materials** (**Supplementary Table 2**) present the ages at diagnosis for the different pathological types.

**Table 1.** **Characteristics of patients with HL**.

Characteristics		Patients with HL (No.)	%
Total		20,423	
Sex			
	Male	11,389	55.8
	Female	9034	44.2
Race			
	White	17,456	85.5
	Black	2052	10.0
	Other^a^	915	4.5
Age at diagnosis (years)			
	0–34	11,777	57.7
	35–64	6502	31.8
	65+	2144	10.5
Year of diagnosis			
	1975–1988	5574	27.2
	1989–2002	6853	33.6
	2003–2018	7996	39.2
Follow-up (months)			
	2–11	1719	8.4
	12–59	4114	20.1
	60–119	3487	17.1
	120–179	3003	14.7
	180+	8100	39.7
Pathological type			
	NSCHL	14,472	70.8
	MCCHL	3688	18.1
	LRCHL	815	4.0
	LDCHL	446	2.2
	NLPHL	1002	4.9

^a^American Indian/AK Native, Asian/Pacific Islander; HL, Hodgkin lymphoma; NSCHL, nodular sclerosing classical HL; MCCHL, mixed-cellularity classical HL; LRCHL, lymphocyte-rich classical HL; LDCHL, lymphocyte-depleted classical HL; NLPHL, nodular lymphocyte-predominant HL.

### 3.2 Mortality Proportion

**Fig. 1.**
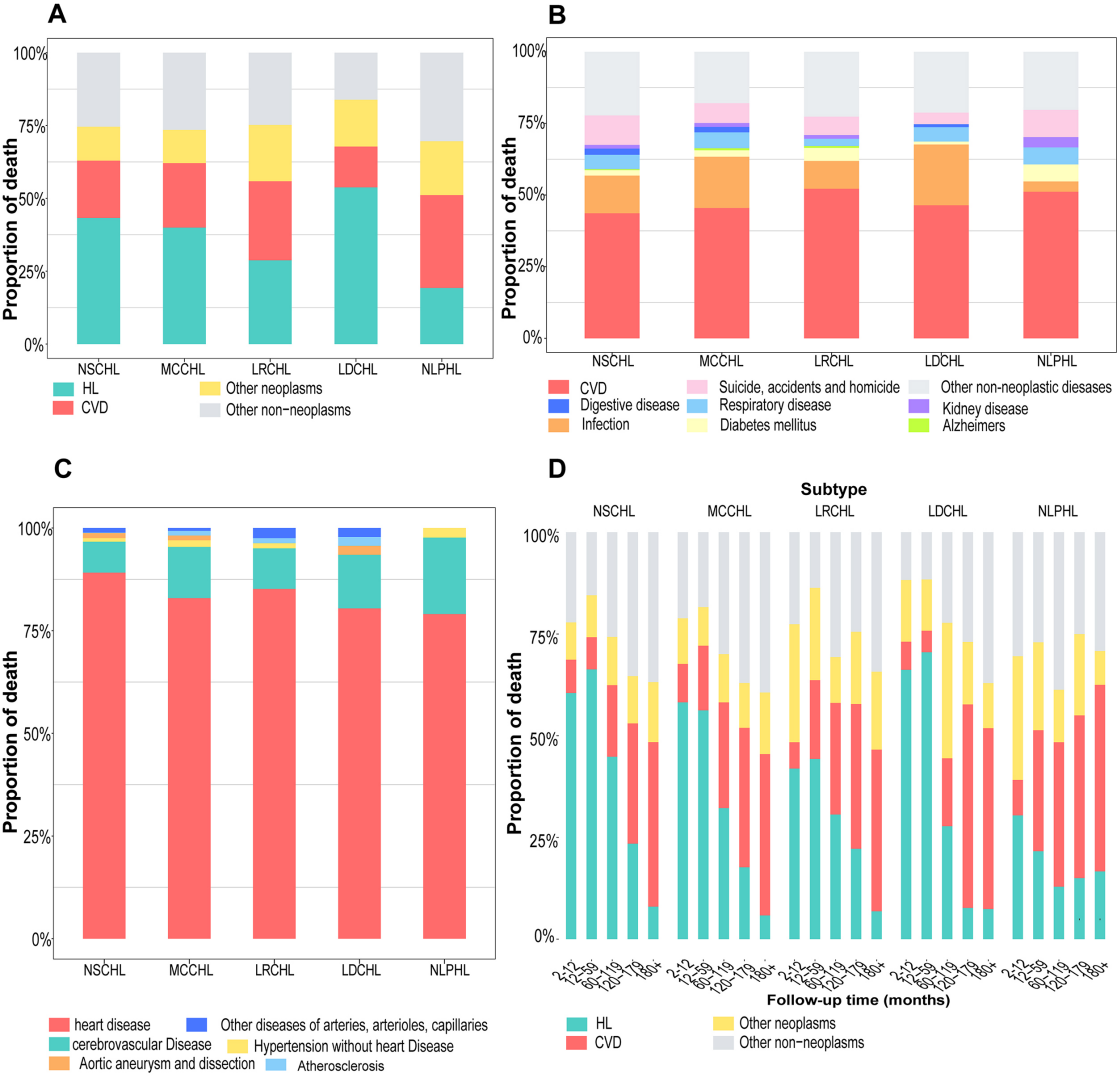
**Proportion of cause-specific death in different HL subtypes**. (A) The proportion of deaths from HL, CVD, other neoplasms, or other non-neoplasms; (B) the proportion of deaths from non-neoplasms; (C) the proportion of deaths from different CVDs; (D) the proportion of cause-specific deaths at different follow-up intervals. HL, Hodgkin lymphoma; CVD, cardiovascular disease; NSCHL, nodular sclerosing classical HL; MCCHL, mixed-cellularity classical HL; LRCHL, lymphocyte-rich classical HL; LDCHL, lymphocyte-depleted classical HL; NLPHL, nodular lymphocyte-predominant HL.

Irrespective of the follow-up interval, CVD PMR exceeded that of HL only in patients with NLPHL. HL remained the predominant cause of death among patients with NSCHL, MCCHL, and LDCHL (Fig. 1A). A similar phenomenon was observed among different types of patients that had been further classified based on age, race, and sex (**Supplementary Fig. 1**). Analysis of cause-specific PMR at different intervals revealed that the proportion of CVD-related deaths exceeded that of HL and, thus, was the leading cause of death among patients with various types. Among NLPHL and other HL subtypes, the proportion of CVD-related deaths surpassed that of HL at 12 and 120 months respectively (Fig. 1D), while patients aged over 64 years with NSCHL observed parallel trends at 60 months of follow-up (**Supplementary Fig. 2**). Meanwhile, CVD was the leading cause of death among all non-neoplastic causes (Fig. 1B). A similar phenomenon was noted among the subpopulations classified according to age, race, and sex (**Supplementary Fig. 3**). Most CVD deaths were due to heart disease (Fig. 1C). Almost all patients aged 0–34 years with MCCHL died from heart disease (**Supplementary Fig. 4**).

### 3.3 Cumulative Cause-specific Mortality

**Fig. 2.**
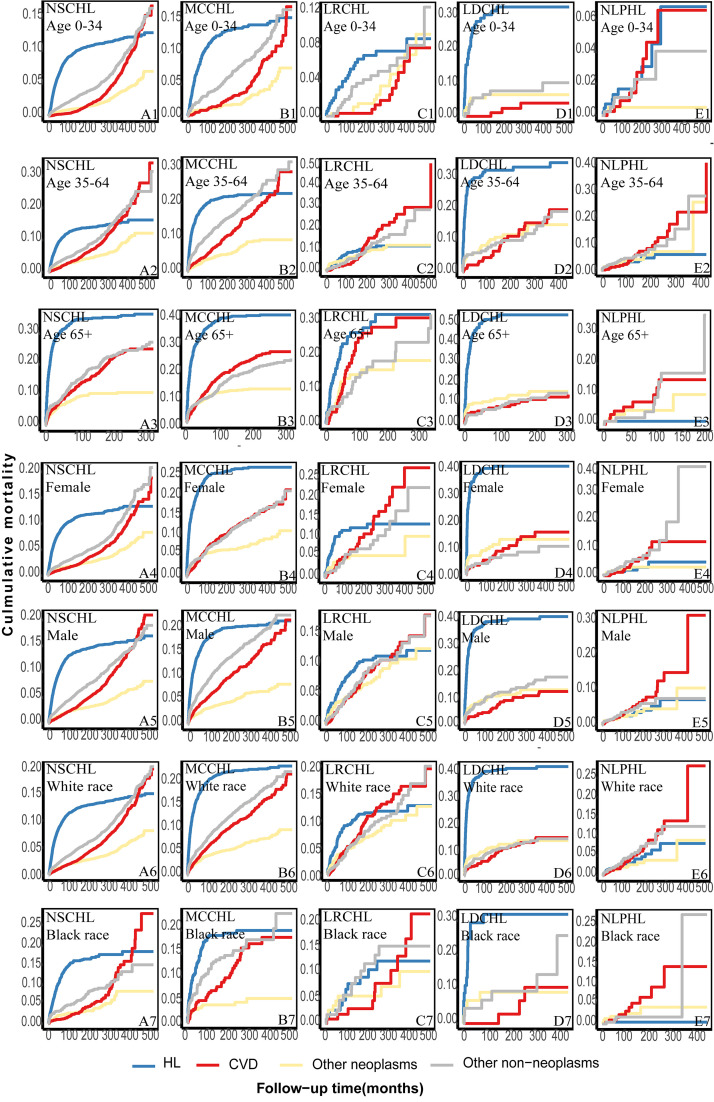
**Further classification of the cumulative cause-specific mortality among different subtypes of patients according to age, race, and sex**. (A) the cumulative cause-specific mortality of patients with NSCHL according to age, race, and sex. (B) the cumulative cause-specific mortality of patients with MCCHL according to age, race, and sex. (C) the cumulative cause-specific mortality of patients with LRCHL according to age, race, and sex. (D) the cumulative cause-specific mortality of patients with LDCHL according to age, race, and sex. (E) the cumulative cause-specific mortality of patients with NLPHL according to age, race, and sex.

Patients with different HL subtypes were further classified according to age, race, and sex. The cumulative HL mortality in all types plateaued, while CVD mortality risks rapidly and continuously increased throughout the follow-up (**Supplementary Fig. 5A–E**). Among patients with NLPHL, the cumulative CVD mortality exceeded that of HL among patients other than those aged 0–34 years old, >64 years old, and those classified as black in race. This was observed among these patients after approximately 100 months of follow-up (Fig. 2E1–7). Among patients with LRCHL, the cumulative mortality from CVD exceeded that of HL among all patients other than those aged 0–34 or more than 64 years after approximately 180 months of follow-up (Fig. 2C1–7). Notably, if patients with MCCHL were treated as a whole, these patients exhibited a higher risk of mortality due to HL than due to CVD throughout follow-up (**Supplementary Fig. 5B**). However, an opposite trend was observed among patients aged 0–34 years after approximately 480 months and aged 35–64 years after approximately 420 months (Fig. 2B1–2). The rest of MCCHL is consistent with the overall trend (Fig. 2B3–7). Among patients with NSCHL, the cumulative mortality from CVD exceeded that from HL among all patients except those aged >64 years. This was observed after approximately 350 months of follow-up among patients aged 35–64 years (Fig. 2A1–7). Patients with LDCHL exhibited no similar temporal trend in CVD mortality (Fig. 2D1–7).

### 3.4 Cumulative CVD Mortality among Different Populations

**Fig. 3.**
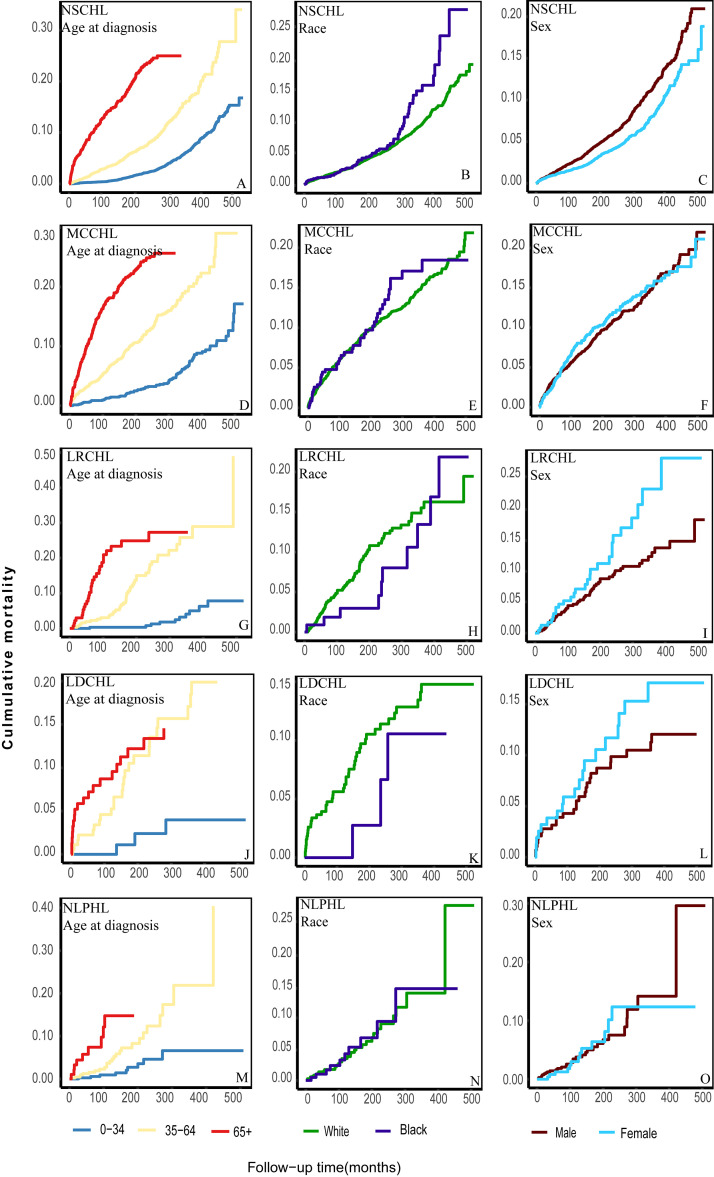
**Further classification of the cumulative CVD mortality among different subtypes of patients according to age, race, and sex**. (A) the cumulative CVD mortality of patients with NSCHL according to age at diagnosis. (B) the cumulative CVD mortality of patients with NSCHL according to race. (C) the cumulative CVD mortality of patients with NSCHL according to sex. (D) the cumulative CVD mortality of patients with MCCHL according to age at diagnosis. (E) the cumulative CVD mortality of patients with MCCHL according to race. (F) the cumulative CVD mortality of patients with MCCHL according to sex. (G) the cumulative CVD mortality of patients with LRCHL according to age at diagnosis. (H) the cumulative CVD mortality of patients with LRCHL according to race. (I) the cumulative CVD mortality of patients with LRCHL according to sex. (J) the cumulative CVD mortality of patients with LDCHL according to age at diagnosis. (K) the cumulative CVD mortality of patients with LDCHL according to race. (L) the cumulative CVD mortality of patients with LDCHL according to sex. (M) the cumulative CVD mortality of patients with NLPHL according to age at diagnosis. (N) the cumulative CVD mortality of patients with NLPHL according to race. (O) the cumulative CVD mortality of patients with NLPHL according to sex.

Patients with HL aged >64 years exhibited the highest risk of CVD mortality, followed by patients aged 35–64 and 0–34 years (Fig. 3A,D,G,J,M); meanwhile, among patients with NSCHL, the cumulative CVD mortality for black patients exceeded that for white patients after approximately 300 months of follow-up (Fig. 3B). Among patients with LDCHL, white patients exhibited a higher risk all the time (Fig. 3K). Cumulative CVD Mortality for MCCHL, LRCHL, and NLPHL did not differ significantly between whites and blacks, respectively (Fig. 3E,H,N). Males diagnosed with NSCHL suffered a higher risk of CVD mortality; a contrary temporal trend was observed among those diagnosed with LRCHL and LDCHL (Fig. 3C,I,L). Meanwhile, cumulative CVD mortality rates for MCCHL and NLPHL were generally consistent in both males and females (Fig. 3F,O).

### 3.5 Cumulative Cause-specific Mortality in Different Periods

**Fig. 4.**
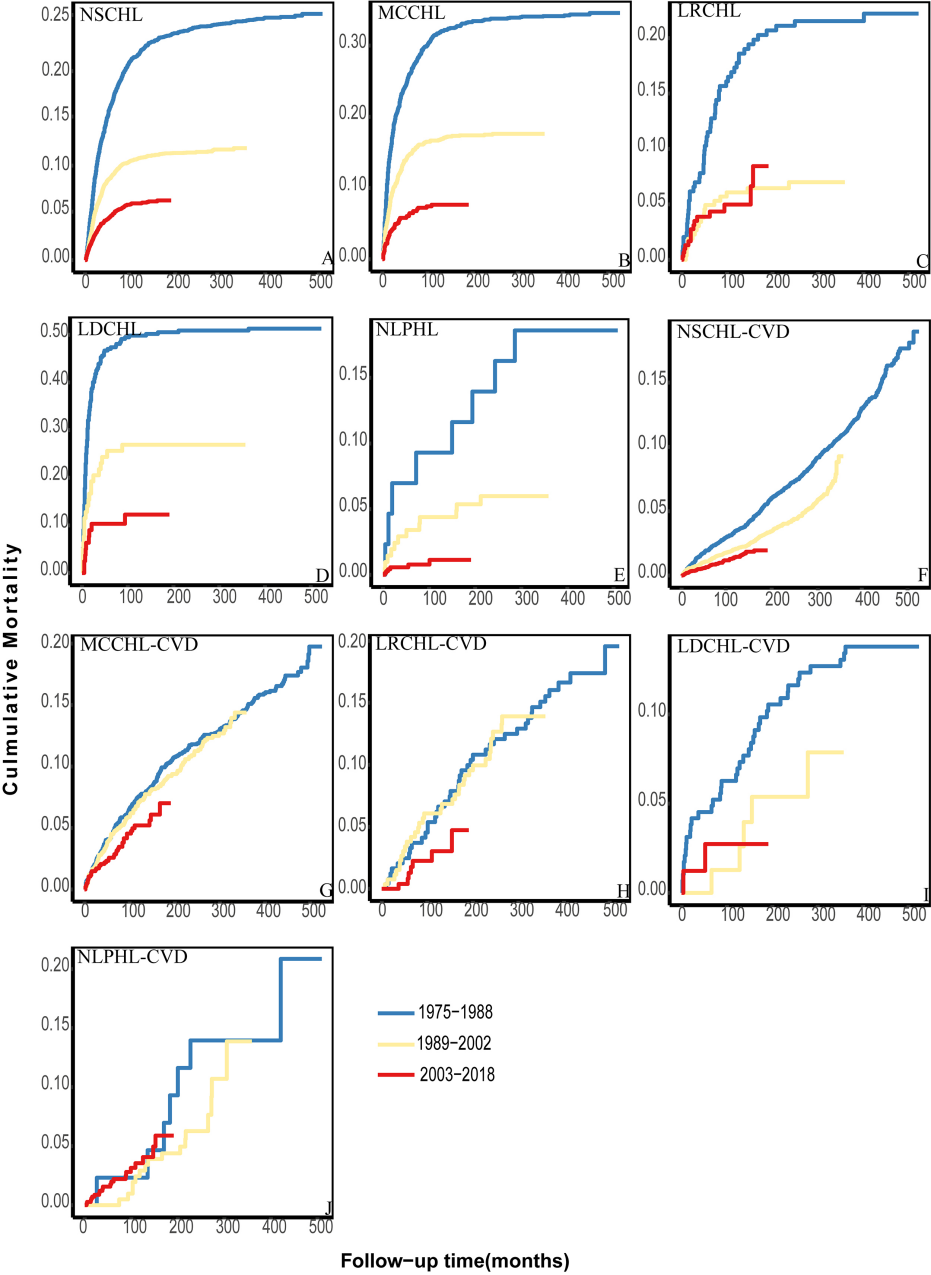
**The cumulative HL and CVD mortality among different patient subtypes in other periods**. (A–E) The cumulative HL mortality over the analyzed date ranges. (F–J) The cumulative CVD mortality of different subtypes over the analyzed date ranges.

Patients in our multicenter cohort were diagnosed between 1975 and 2018. These 44 years were divided into three consecutive periods according to the year of diagnosis (1975–1988; 1989–2002; 2003–2018). Among patients with NSCHL, MCCHL, LDCHL, and NLPHL, the cumulative HL mortality significantly decreased from 1975–1998 to 2003–2018 (Fig. 4A,B,D,E). No improvement was observed among patients with LRCHL diagnosed between 2003–2018 and 1989–2002 (Fig. 4C). Over these four decades, cumulative CVD mortality decreased slowly among patients with NSCHL, MCCHL, and LRCHL (Fig. 4F–H) and did not change among patients with LDCHL and NLPHL (Fig. 4I,J).

Patients with different HL subtypes were further divided into various subpopulations according to age, race, and sex. Among all subpopulations, the cumulative HL mortality decreased significantly in a temporal manner (**Supplementary Figs. 6,7,8**). The cumulative CVD mortality decreased slowly among most patients (**Supplementary Figs. 9,10,11**). No changes were observed among patients with NSCHL aged 0–34 years (**Supplementary Fig. 9A**), black patients with MCCHL (**Supplementary Fig. 10D**), or female patients with NSCHL (**Supplementary Fig. 11B**) between 1989–2002 and 2003–2018. Among white patients with NLPHL, the risk in the 2003–2018 period exceeded that in the 1989–2002 period from the beginning of follow-up (**Supplementary Fig. 10H**).

### 3.6 CVD Mortality among Patients with HL Compared with the General Population

SMR and AER for CVD were assessed at different follow-up intervals for all HL subtypes. All patients with HL exhibited a higher CVD mortality rate than the general population over time. However, only patients with MCCHL suffered a higher CVD mortality at all time points compared with the general population. SMR significantly differed among patients diagnosed with various HL subtypes over the same follow-up interval. During the first interval, the SMR for CVD among patients with MCCHL reached 5.79 (95% CI: 4.18–7.83) and was higher than among other types. At the 120–179 months interval, patients with LDCHL exhibited the highest SMR (SMR = 9.27; 95% CI: 4.93–15.85) and a corresponding AER of 209 (Table 2).

**Table 2.** **Standardized mortality ratio, absolute excess risk, and proportional mortality ratio for CVD among patients with HL at different follow-up intervals**.

Histologic type	Follow-up interval (months)	No.	SMR	95% CI	AER	PMR (%)
NSCHL						
	2–11	50	1.69	1.26–2.23	17.45	8.20
	12–59	109	0.89	0.73–1.08	–2.68	7.88
	60–119	126	1.04	0.87–1.24	1.12	17.60
	120–179	122	1.32	1.10–1.58	8.15	29.54
	180+	404	2.55	2.31–2.82	39.24	40.40
MCCHL						
	2–11	42	5.79	4.18–7.83	121.14	9.44
	12–59	102	3.69	3.01–4.48	68.02	15.89
	60–119	92	3.57	2.88–4.38	64.87	25.99
	120–179	62	3.26	2.50–4.18	57.05	34.25
	180+	136	3.7	3.11–4.38	68.27	39.65
LRCHL						
	2–11	2	1.20	0.14–4.33	5.06	6.45
	12–59	17	2.44	1.42–3.91	36.38	19.32
	60–119	17	2.54	1.48–4.07	38.92	27.42
	120–179	16	3.24	1.85–5.26	56.53	35.56
	180+	29	3.17	2.12–4.56	54.83	39.73
LDCHL						
	2–11	10	4.71	2.26–8.66	93.78	6.90
	12–59	5	1.57	0.51–3.65	14.29	5.26
	60–119	6	2.32	0.85–5.04	33.25	16.67
	120–179	13	9.27	4.93–15.85	209	50.00
NLPHL						
	2–11	2	0.9	0.10–3.24	–2.56	8.70
	12–59	11	1.4	0.70–2.50	10	29.73
	60–119	11	1.67	0.83–2.99	17	35.48
	120–179	8	2.13	0.92–4.20	28.54	40.00
	180+	11	3.34	1.67–5.98	59.17	45.83

SMR, standardized mortality ratio; AER, absolute excess risk; CI, confidence interval; PMR, proportional mortality ratio.

## 4. Discussion

Herein, the cumulative HL mortality decreased rapidly over the past decades regardless of the HL subtype, which means HL treatment has substantially improved. However, we found that the risk of CVD mortality decreased slowly or remained unchanged. From the initial diagnosis to >500 months of follow-up, the cumulative CVD mortality increased continually without a plateau and exceeded that of HL at different times in most patients with various HL subtypes. Additionally, black patients or males with NSCHL exhibited a higher CVD mortality risk, whereas a contrary trend was observed among those with LRCHL or LDCHL. The temporal trend of CVD mortality risk presented disparities among survivors of various HL subtypes.

Previous studies, without regard to the different HL subtypes, reported that HL remained the first cause of death and CVD was only one of the significant causes of death among long-term survivors of HL [8, 16, 17, 27, 28, 29, 30]. Herein, we found that the proportion of CVD-related deaths exceeded that of HL-related deaths among patients with NLPHL. The studies mentioned above did not examine the proportion of cause-specific deaths at different follow-up intervals. When considering the follow-up intervals, we found that the proportion of CVD-related deaths exceeded that of HL over time in patients with various types. However, the CVD mortality risk in patients with HL could have been underestimated in the past. The time when the proportion of CVD-related deaths exceeded that of HL was earlier in patients with NLPHL than in those with other types. Although patients with NLPHL accounted for a small portion of the overall HL population, further studies should focus on them. Our results provide a more detailed reflection of the CVD-related deaths among patients with HL. Researchers should consider HL subtypes when assessing the risk of CVD mortality among patients with HL.

We examined the temporal trend in cumulative CVD mortality among patients with different HL subtypes. As age, race, and sex are risk factors for CVD morbidity and mortality among patients with HL, patients diagnosed with different HL subtypes were further classified into appropriate subpopulations [22, 31, 32, 33]. Previous studies reported that the CVD mortality risk increased as follow-up time progressed [17, 29, 34, 35]. This trend occurred in various forms. However, inconsistent with previous studies, our study, which used more recent data, was the first to demonstrate that the cumulative CVD mortality exceeded that of HL at different times in patients with different HL types [8, 31, 34, 35]. For instance, the cumulative CVD mortality exceeded that of HL among patients with NLPHL, LRCHL, MCCHL, and NSCHL aged 35–64 years after approximately 100, 180, 420, and 350 months of follow-up, respectively. These results demonstrated that the temporal trend in CVD mortality risk exhibited significant disparities in various HL subtypes. Indeed, cumulative CVD exceeded that of HL because the cumulative HL mortality has decreased sharply over previous decades [36, 37, 38]. We observed that HL mortality risk has declined sharply over the past decades. Additionally, the CVD mortality risk was uncontrolled. Our results demonstrate that cumulative CVD mortality has decreased slowly over the three consecutive periods. Indeed, the mortality of patients with LDCHL and NLPHL has remained unchanged. CVD became the leading cause of death as the follow-up progressed. Thus, patients should take more measures to reduce the risk of CVD mortality.

The time when PMR and cumulative CVD mortality exceeded that of HL among patients with NLPHL was the shortest compared to patients with other HL subtypes. Since NLPHL was more indolent over its course, observation is an important option [38, 39]. In theory, those patients might suffer less CVD toxicity from chemotherapy and radiotherapy treatments than patients with other subtypes. This phenomenon occurred for decreased chemotherapy and radiotherapy dosage and warrants further research as it is likely caused by several mechanisms, such as the administration of cardioprotective drugs or surgical treatment during treatment [20, 40]. Strategies for reducing the risk of CVD mortality should not be limited to adjusting the chemotherapy dose or developing new therapeutic approaches whose adverse effects on the cardiovascular system are unclear in the short term.

Consistent with previous studies, older individuals had a higher risk of CVD mortality [23, 30]. Different from other studies without regard to the HL subtypes, not all black or male patients exhibited a higher CVD mortality risk. Black or male patients with NSCHL exhibited a higher CVD mortality risk, whereas a contrary trend was observed among those with LRCHL or LDCHL. This may be related to sex chromosomes and hormone levels. Variations in socioeconomic status and living conditions may contribute to disparities observed across different racial and ethnic groups[41, 42]. However, the specific reasons need to be further studied [43]. We might need to focus on these disparities in CVD mortality risk in the process of HL treatment.

CVD mortality among patients with any HL subtype exceeded that of the general population over time (Table 2) [18]. The temporal trend of SMR was significantly different among patients with different HL subtypes. Additionally, SMR and AER similarly differed over the same interval among patients with different HL subtypes. We suspect that patients with HL suffering from those subtypes with higher SMR and AER likely suffer higher levels of CVD toxicity in the setting of the same chemotherapy and radiotherapy dosage and regimen.

The PMR for CVD in different subtypes demonstrated various temporal trends throughout the follow-up. Although the cumulative mortality of CVD exceeded that of HL in many survivors of different HL subtypes, the period this event happened differed for the various types. Furthermore, the phenomenon that the cumulative mortality of CVD exceeded that of HL did not occur in every type among patients of the same age, race, or sex. All these disparities indicate that we might need to take measures depending on subtypes. For example, earlier measures might be used to reduce CVD mortality in populations where the risk of CVD mortality exceeded that of HL. We might need to emphasize female or white patients for some types of HL rather than only focusing on male or black patients who were considered to be at higher risk in previous studies. However, different histological subtypes have not currently been translated into different treatment approaches.

## 5. Limitations

There are limitations to this study. First, we only performed an epidemiological study on patients with different HL subtypes. This study only reflected the epidemiological situation of CVD for the survivors of different HL types and did not reveal the reasons for this phenomenon. We believe that many factors, including smoking, obesity, chemotherapy, and radiotherapy, can result in disparities in epidemiological studies for CVD. It is essential to determine the reasons for further reducing the risk of CVD mortality. For example, if the high dose of chemotherapy resulted in the phenomenon that the risk of CVD mortality exceeded that of HL and happened earlier in some populations than others, we might be able to take measures to balance the chemotherapy and the risk of CVD mortality in these populations. If smoking or a lack of monitoring of other CVD risk factors resulted in a higher CVD mortality risk than HL, we could strengthen supervision in those populations. Regrettably, information on treatment options for HL associated with cardiovascular complications, including chemotherapy drug dose and radiotherapy site, was missing from the SEER database. This limits further analysis of their impact on the risk of CVD death in patients with HL [34, 44, 45, 46, 47, 48]. Information on monitoring for cardiac complications is useful. Second, statistical bias and residual confounding are inevitable, as this is a retrospective analysis of an extensive database. We will further conduct studies to determine the reasons for the disparities in the epidemiological situation of CVD in the future. Third, because the SEER database did not provide information on subclinical cardiovascular diseases, cardiovascular comorbidities, or common risk factors, we could not describe the CVD mortality risk in the HL subgroup with these influencing factors [49]. Further studies on heart diseases, including coronary heart disease, valvular disease, and heart failure, could help determine targeted measures to decrease mortality due to specific diseases.

## 6. Conclusions

Over the past decades, the HL mortality risk has decreased rapidly; however, the CVD mortality risk has reduced slowly or remained unchanged. The CVD mortality risk for patients with various types exceeded HL at different times as follow-up progressed. The temporal trend of CVD mortality risk exhibited significant disparities among patients with various HL subtypes. Patients should take more measures to control the CVD mortality risk, depending on their HL types.

## Data Availability

The datasets are publicly available from the SEER database (http://seer.cancer.gov).

## Abbreviations

CVD, cardiovascular disease; HL, Hodgkin lymphoma; SMR, standardized mortality ratio; AER, absolute excess risk; NLPHL, nodular lymphocyte-predominant HL; NSCHL, nodular sclerosing classical HL; MCCHL, mixed-cellularity classical HL; LRCHL, lymphocyte-rich classical HL; LDCHL, lymphocyte-depleted classical HL.

## Supplementary Material

Supplementary material associated with this article can be found, in the online version, at https://doi.org/10.31083/RCM24981.

## References

[1] (1832). On some Morbid Appearances of the Absorbent Glands and Spleen. *Medico-chirurgical Transactions*.

[2] Jacob A, Thyagarajan B, Kumar MP, Shaikh N, Sharon D (2018). Cardiovascular effects of Hodgkin’s lymphoma: a review of literature. *Journal of Cancer Research and Clinical Oncology*.

[3] Bray F, Laversanne M, Sung H, Ferlay J, Siegel RL, Soerjomataram I (2024). Global cancer statistics 2022: GLOBOCAN estimates of incidence and mortality worldwide for 36 cancers in 185 countries. *CA: a Cancer Journal for Clinicians*.

[4] Weniger MA, Küppers R (2021). Molecular biology of Hodgkin lymphoma. *Leukemia*.

[5] Paviglianiti A, Rampi N (2023). Advances and Clinical Outcomes in Hodgkin Lymphoma in the Era of Novel Therapies. *Journal of Clinical Medicine*.

[6] Applefeld MM, Cole JF, Pollock SH, Sutton FJ, Slawson RG, Singleton RT (1981). The late appearance of chronic pericardial disease in patients treated by radiotherapy for Hodgkin’s disease. *Annals of Internal Medicine*.

[7] Adams MJ, Constine LS, Lipshultz SE (2007). Late effects of therapy for Hodgkin’s lymphoma. *Current Hematologic Malignancy Reports*.

[8] de Vries S, Schaapveld M, Janus CPM, Daniëls LA, Petersen EJ, van der Maazen RWM (2021). Long-Term Cause-Specific Mortality in Hodgkin Lymphoma Patients. *Journal of the National Cancer Institute*.

[9] Sklar C, Whitton J, Mertens A, Stovall M, Green D, Marina N (2000). Abnormalities of the thyroid in survivors of Hodgkin’s disease: data from the Childhood Cancer Survivor Study. *The Journal of Clinical Endocrinology and Metabolism*.

[10] Chen L, Zheng Y, Yu K, Chen S, Wang W, Gale RP (2022). Changing causes of death in persons with haematological cancers 1975-2016. *Leukemia*.

[11] Núñez-García B, Clemente MB, Sánchez JC, Royuela A, Ibargüen BCSD, Méndez M (2023). Long-term outcomes in Hodgkin lymphoma survivors. Temporary trends and comparison with general population. *Hematological Oncology*.

[12] Hull MC, Morris CG, Pepine CJ, Mendenhall NP (2003). Valvular dysfunction and carotid, subclavian, and coronary artery disease in survivors of hodgkin lymphoma treated with radiation therapy. *JAMA*.

[13] Heidenreich PA, Hancock SL, Vagelos RH, Lee BK, Schnittger I (2005). Diastolic dysfunction after mediastinal irradiation. *American Heart Journal*.

[14] Mulrooney DA, Yeazel MW, Kawashima T, Mertens AC, Mitby P, Stovall M (2009). Cardiac outcomes in a cohort of adult survivors of childhood and adolescent cancer: retrospective analysis of the Childhood Cancer Survivor Study cohort. *BMJ (Clinical Research Ed.)*.

[15] van Nimwegen FA, Ntentas G, Darby SC, Schaapveld M, Hauptmann M, Lugtenburg PJ (2017). Risk of heart failure in survivors of Hodgkin lymphoma: effects of cardiac exposure to radiation and anthracyclines. *Blood*.

[16] Gao J, Chen Y, Wu P, Wang F, Tao H, Shen Q (2021). Causes of death and effect of non-cancer-specific death on rates of overall survival in adult classic Hodgkin lymphoma: a populated-based competing risk analysis. *BMC Cancer*.

[17] Aleman BMP, van den Belt-Dusebout AW, Klokman WJ, Van’t Veer MB, Bartelink H, van Leeuwen FE (2003). Long-term cause-specific mortality of patients treated for Hodgkin’s disease. *Journal of Clinical Oncology: Official Journal of the American Society of Clinical Oncology*.

[18] Zhang H, He M, Zhang P, Gao Y, Ouyang L, He X (2024). Long-term risks of cardiovascular death among older patients with major hematological malignancies: a population-based cohort study from SEER database. *Cancer Epidemiology, Biomarkers & Prevention: a Publication of the American Association for Cancer Research, Cosponsored by the American Society of Preventive Oncology*.

[19] Lu Z, Teng Y, Ning X, Wang H, Feng W, Ou C (2022). Long-term risk of cardiovascular disease mortality among classic Hodgkin lymphoma survivors. *Cancer*.

[20] Brice P, de Kerviler E, Friedberg JW (2021). Classical Hodgkin lymphoma. *Lancet (London, England)*.

[21] de Vries S, Schaapveld M, van Nimwegen FA, Jóźwiak K, Lugtenburg PJ, Daniëls LA (2018). High burden of subsequent malignant neoplasms and cardiovascular disease in long-term Hodgkin lymphoma survivors. *British Journal of Cancer*.

[22] Bhakta N, Liu Q, Yeo F, Baassiri M, Ehrhardt MJ, Srivastava DK (2016). Cumulative burden of cardiovascular morbidity in paediatric, adolescent, and young adult survivors of Hodgkin’s lymphoma: an analysis from the St Jude Lifetime Cohort Study. *The Lancet. Oncology*.

[23] Amini A, Murphy B, Cost CR, Garrington TP, Greffe BS, Liu AK (2016). Cardiac Mortality in Children and Adolescents with Hodgkin’s Lymphoma: A Surveillance, Epidemiology and End Results Analysis. *Journal of Adolescent and Young Adult Oncology*.

[24] National Cancer Institute (1973). About the SEER program. https://seer.cancer.gov/about/.

[25] Guan T, Monteiro O, Chen D, Luo Z, Chi K, Li Z (2024). Long-term and short-term cardiovascular disease mortality among patients of 21 non-metastatic cancers. *Journal of Advanced Research*.

[26] Du B, Wang F, Wu L, Wang Z, Zhang D, Huang Z (2021). Cause-specific mortality after diagnosis of thyroid cancer: a large population-based study. *Endocrine*.

[27] Mauch PM, Kalish LA, Marcus KC, Shulman LN, Krill E, Tarbell NJ (1995). Long-term survival in Hodgkin’s disease relative impact of mortality, second tumors, infection, and cardiovascular disease. *The Cancer Journal from Scientific American*.

[28] Mertens AC, Yasui Y, Neglia JP, Potter JD, Nesbit ME, Ruccione K (2001). Late mortality experience in five-year survivors of childhood and adolescent cancer: the Childhood Cancer Survivor Study. *Journal of Clinical Oncology: Official Journal of the American Society of Clinical Oncology*.

[29] Kiserud CE, Loge JH, Fosså A, Holte H, Cvancarova M, Fosså SD (2010). Mortality is persistently increased in Hodgkin’s lymphoma survivors. *European Journal of Cancer (Oxford, England: 1990)*.

[30] Al-Kindi SG, Abu-Zeinah GF, Kim CH, Hejjaji V, William BM, Caimi PF (2015). Trends and Disparities in Cardiovascular Mortality Among Survivors of Hodgkin Lymphoma. *Clinical Lymphoma, Myeloma & Leukemia*.

[31] van Nimwegen FA, Schaapveld M, Janus CPM, Krol ADG, Petersen EJ, Raemaekers JMM (2015). Cardiovascular disease after Hodgkin lymphoma treatment: 40-year disease risk. *JAMA Internal Medicine*.

[32] Castellino SM, Geiger AM, Mertens AC, Leisenring WM, Tooze JA, Goodman P (2011). Morbidity and mortality in long-term survivors of Hodgkin lymphoma: a report from the Childhood Cancer Survivor Study. *Blood*.

[33] Kahn JM, Kelly KM, Pei Q, Bush R, Friedman DL, Keller FG (2019). Survival by Race and Ethnicity in Pediatric and Adolescent Patients With Hodgkin Lymphoma: A Children’s Oncology Group Study. *Journal of Clinical Oncology: Official Journal of the American Society of Clinical Oncology*.

[34] Hudson MM, Poquette CA, Lee J, Greenwald CA, Shah A, Luo X (1998). Increased mortality after successful treatment for Hodgkin’s disease. *Journal of Clinical Oncology: Official Journal of the American Society of Clinical Oncology*.

[35] Ng AK, Bernardo MP, Weller E, Backstrand KH, Silver B, Marcus KC (2002). Long-term survival and competing causes of death in patients with early-stage Hodgkin’s disease treated at age 50 or younger. *Journal of Clinical Oncology: Official Journal of the American Society of Clinical Oncology*.

[36] Xavier AC, Epperla N, Taub JW, Costa LJ (2018). Excess mortality among 10-year survivors of classical Hodgkin lymphoma in adolescents and young adults. *American Journal of Hematology*.

[37] Armstrong GT, Yasui Y, Robison LL (2016). Reduction in Late Mortality after Childhood Cancer. *The New England Journal of Medicine*.

[38] Shanbhag S, Ambinder RF (2018). Hodgkin lymphoma: A review and update on recent progress. *CA: a Cancer Journal for Clinicians*.

[39] Eichenauer DA, Engert A (2020). How I treat nodular lymphocyte-predominant Hodgkin lymphoma. *Blood*.

[40] Eichenauer DA, Hartmann S (2023). Nodular lymphocyte-predominant Hodgkin lymphoma: current management strategies and evolving approaches to individualize treatment. *Expert Review of Hematology*.

[41] Chi K, Zhou R, Luo Z, Zhao H, Jiang Y, He B (2023). Non-cancer-specific survival in patients with primary central nervous system lymphoma: A multi-center cohort study. *Frontiers in Oncology*.

[42] Kim SY, Song HK, Lee SK, Kim SG, Woo HG, Yang J (2020). Sex-Biased Molecular Signature for Overall Survival of Liver Cancer Patients. *Biomolecules & Therapeutics*.

[43] Pulido JS, Vierkant RA, Olson JE, Abrey L, Schiff D, O’Neill BP (2009). Racial differences in primary central nervous system lymphoma incidence and survival rates. *Neuro-oncology*.

[44] Cutter DJ, Ramroth J, Diez P, Buckle A, Ntentas G, Popova B (2021). Predicted Risks of Cardiovascular Disease Following Chemotherapy and Radiotherapy in the UK NCRI RAPID Trial of Positron Emission Tomography-Directed Therapy for Early-Stage Hodgkin Lymphoma. *Journal of Clinical Oncology: Official Journal of the American Society of Clinical Oncology*.

[45] Maraldo MV, Giusti F, Vogelius IR, Lundemann M, van der Kaaij MAE, Ramadan S (2015). Cardiovascular disease after treatment for Hodgkin’s lymphoma: an analysis of nine collaborative EORTC-LYSA trials. *The Lancet. Haematology*.

[46] Hancock SL, Tucker MA, Hoppe RT (1993). Factors affecting late mortality from heart disease after treatment of Hodgkin’s disease. *JAMA*.

[47] Wang L, Wang F, Chen L, Geng Y, Yu S, Chen Z (2021). Long-term cardiovascular disease mortality among 160 834 5-year survivors of adolescent and young adult cancer: an American population-based cohort study. *European Heart Journal*.

[48] Strongman H, Gadd S, Matthews A, Mansfield KE, Stanway S, Lyon AR (2019). Medium and long-term risks of specific cardiovascular diseases in survivors of 20 adult cancers: a population-based cohort study using multiple linked UK electronic health records databases. *Lancet (London, England)*.

[49] Guan T, Jiang Y, Luo Z, Liang Y, Feng M, Lu Z (2023). Long-term risks of cardiovascular death in a population-based cohort of 1,141,675 older patients with cancer. *Age and Ageing*.

