# Supplementary material for: Cardiovascular Disease Mortality Risk among Long-term Survivors of Different Hodgkin Lymphoma Types
Source: Rev Cardiovasc Med. 2025 Jun 30;26(6):24981. doi: 10.31083/RCM24981 (PMC12230821; doi:10.31083/RCM24981)
Supplement: Supplementary file 1 [file 2153-8174-26-6-24981-s1.pdf]

Journal name：***Reviews in Cardiovascular Medicine***

Table S1. The international Classification of Disease-10 code for cardiovascular diseases

Disease	International Classification of Disease-10 code
heart disease	I00–I09, I11, I13, I20–I51
cerebrovascular disease	I60–I69
atherosclerosis	I70
hypertension without heart disease	I10, I12
aortic aneurysm and dissection	I71
other diseases of arteries, arterioles, and capillaries	I72–I78

Table S2. Ages at diagnosis for the different pathological types

Variable	No.(%)				
	NSCHL	MCCHL	LRCHL	LDCHL	NLPHL
Age at diagnosis (years)					
0-34	9426(65.1%)	1461(39.6%)	345(42.3%)	110(24.7%)	435(43.4%)
35-64	4063(28.1%)	1455(39.5%)	332(40.8%)	182(40.8%)	470(46.9%)
65+	983(6.8%)	772(20.9%)	138(16.9%)	154(34.5%)	97(9.7%)

HL Hodgkin lymphoma, cHL classical Hodgkin lymphoma, NSCHL nodular sclerosis cHL, MCCHL mixed-cellularity cHL, LRCHL lymphocyte-rich cHL, LDCHL lymphocyte-depleted cHL, NLPHL nodular lymphocyte-predominant HL.

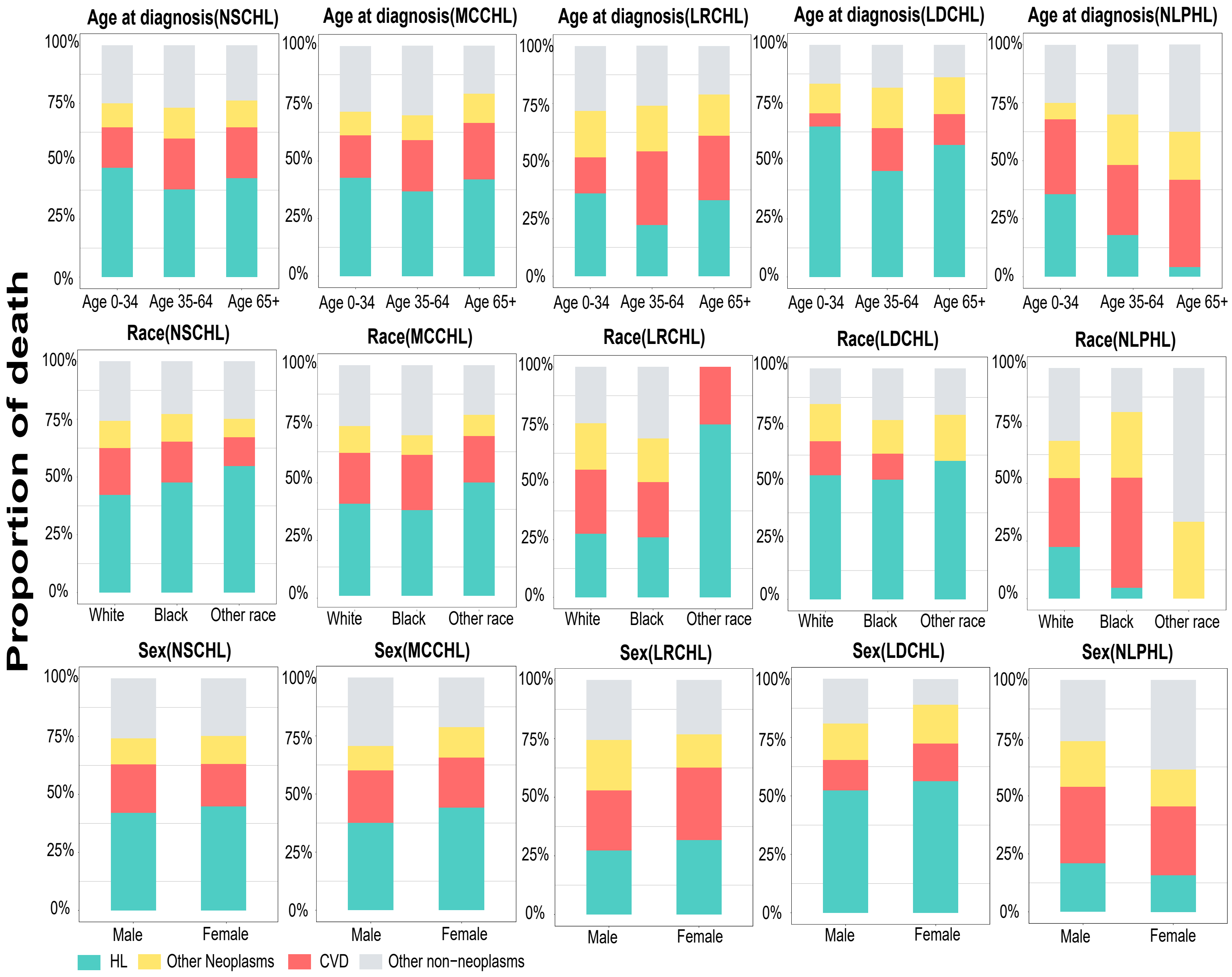

Figure S1. Proportion of cause-specific death among different subtypes of patients further classified according to age, race, sex. HL Hodgkin lymphoma, CVD cardiovascular disease, cHL classical Hodgkin lymphoma, NSCHL nodular sclerosis cHL, MCCHL mixed-cellularity cHL, LRCHL lymphocyte-rich cHL, LDCHL lymphocyte-depleted cHL, NLPHL nodular lymphocyte-predominant HL.

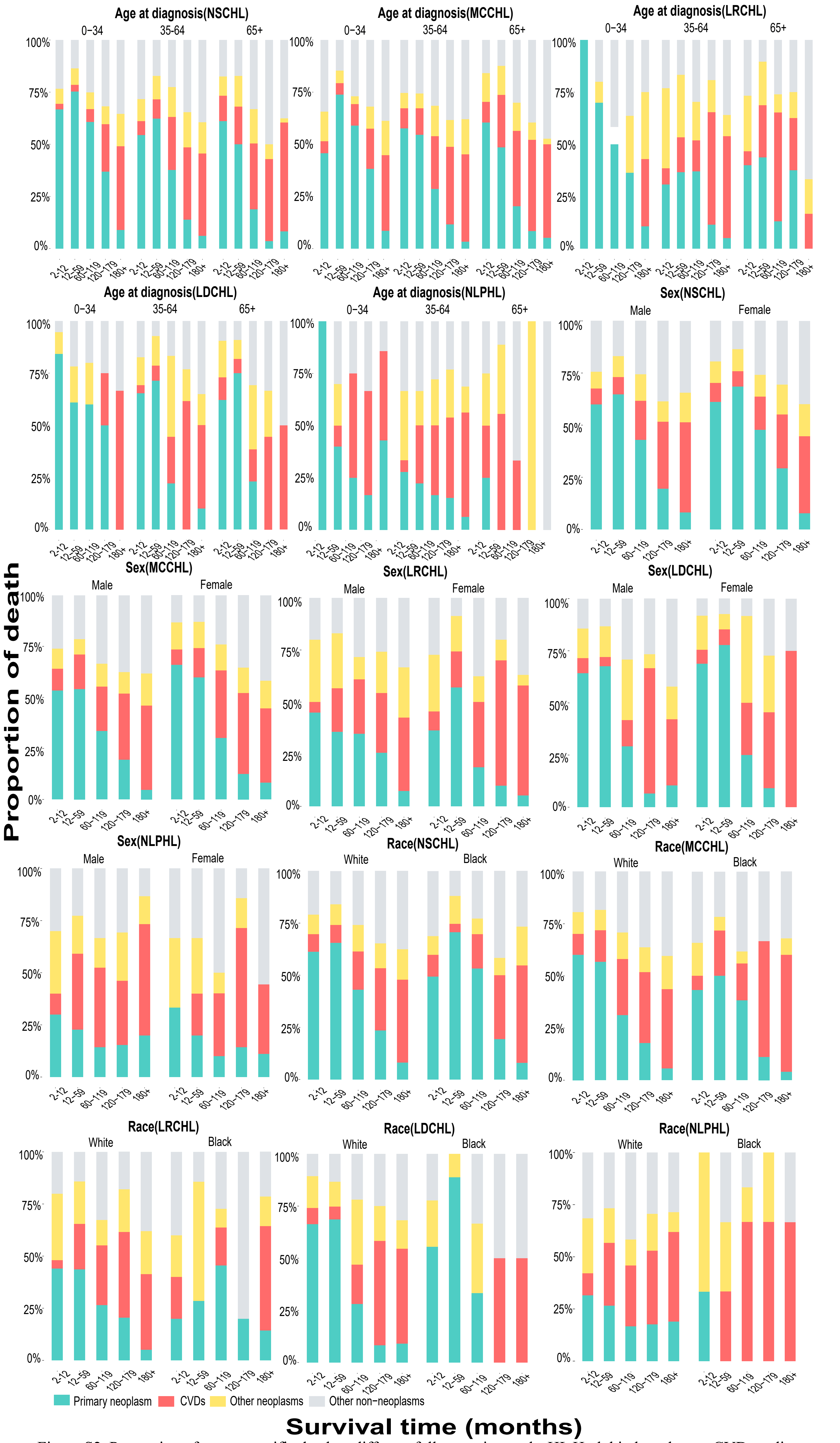

Figure S2. Proportion of cause-specific death at different follow-up intervals. HL Hodgkin lymphoma, CVD cardiovascular disease, cHL classical Hodgkin lymphoma, NSCHL nodular sclerosis cHL, MCCHL mixed-cellularity cHL, LRCHL lymphocyte-rich cHL, LDCHL lymphocyte-depleted cHL, NLPHL nodular lymphocyte-predominant HL.

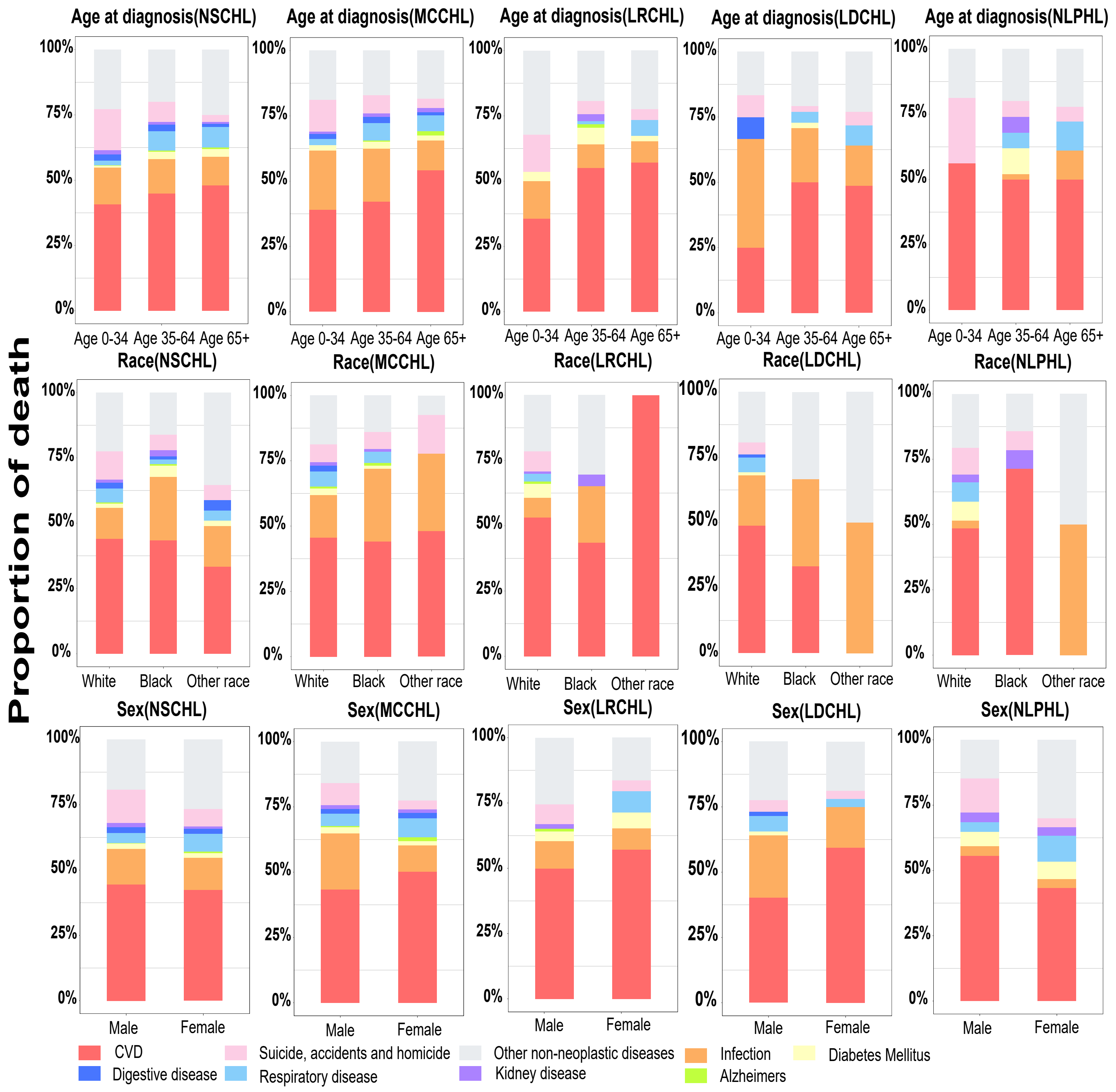

Figure S3. Proportion of non-neoplasms death among different subtypes of patients further classified according to age, race, sex. HL Hodgkin lymphoma, CVD cardiovascular disease, cHL classical Hodgkin lymphoma, NSCHL nodular sclerosis cHL, MCCHL mixed-cellularity cHL, LRCHL lymphocyte-rich cHL, LDCHL lymphocyte-depleted cHL, NLPHL nodular lymphocyte-predominant HL.

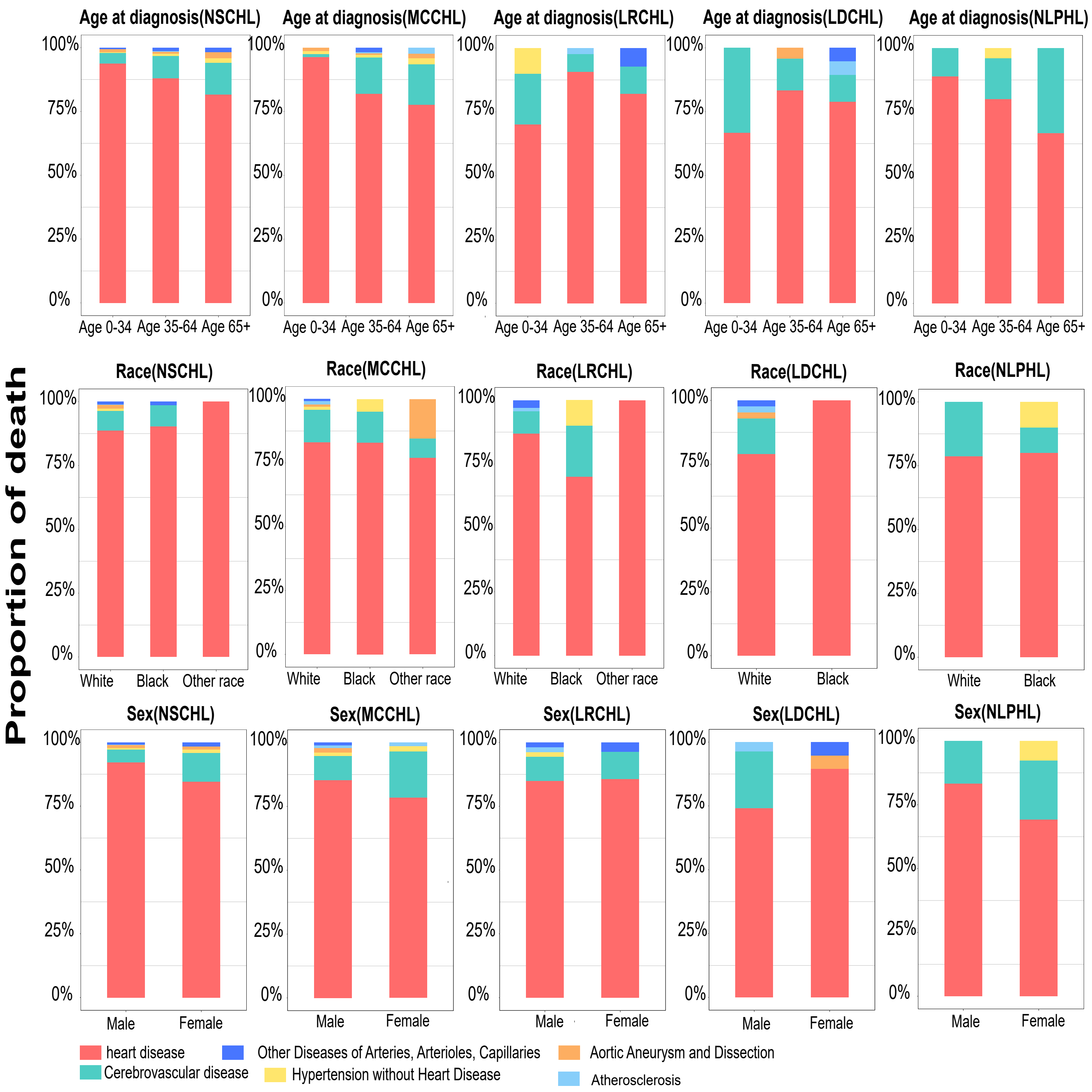

Figure S4. Proportion of CVD death among different subtypes of patients further classified according to age, race, sex. HL Hodgkin lymphoma, CVD cardiovascular disease, cHL classical Hodgkin lymphoma, NSCHL nodular sclerosis cHL, MCCHL mixed-cellularity cHL, LRCHL lymphocyte-rich cHL, LDCHL lymphocyte-depleted cHL, NLPHL nodular lymphocyte-predominant HL.

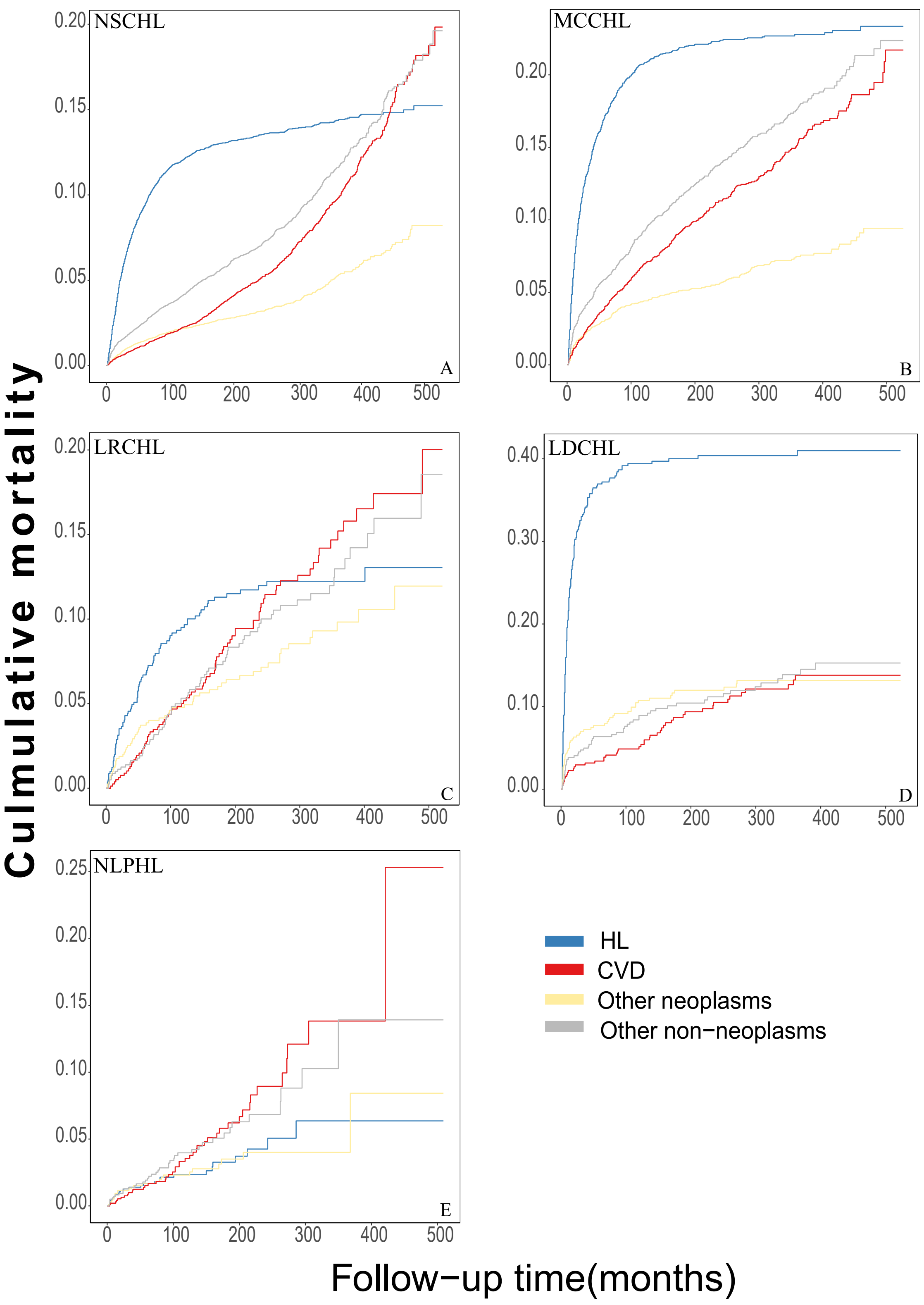

Figure S5. The cumulative cause-specific mortality in different subtypes. HL Hodgkin lymphoma, CVD cardiovascular disease, cHL classical Hodgkin lymphoma, NSCHL nodular sclerosis cHL, MCCHL mixed-cellularity cHL, LRCHL lymphocyte-rich cHL, LDCHL lymphocyte-depleted cHL, NLPHL nodular lymphocyte-predominant HL.

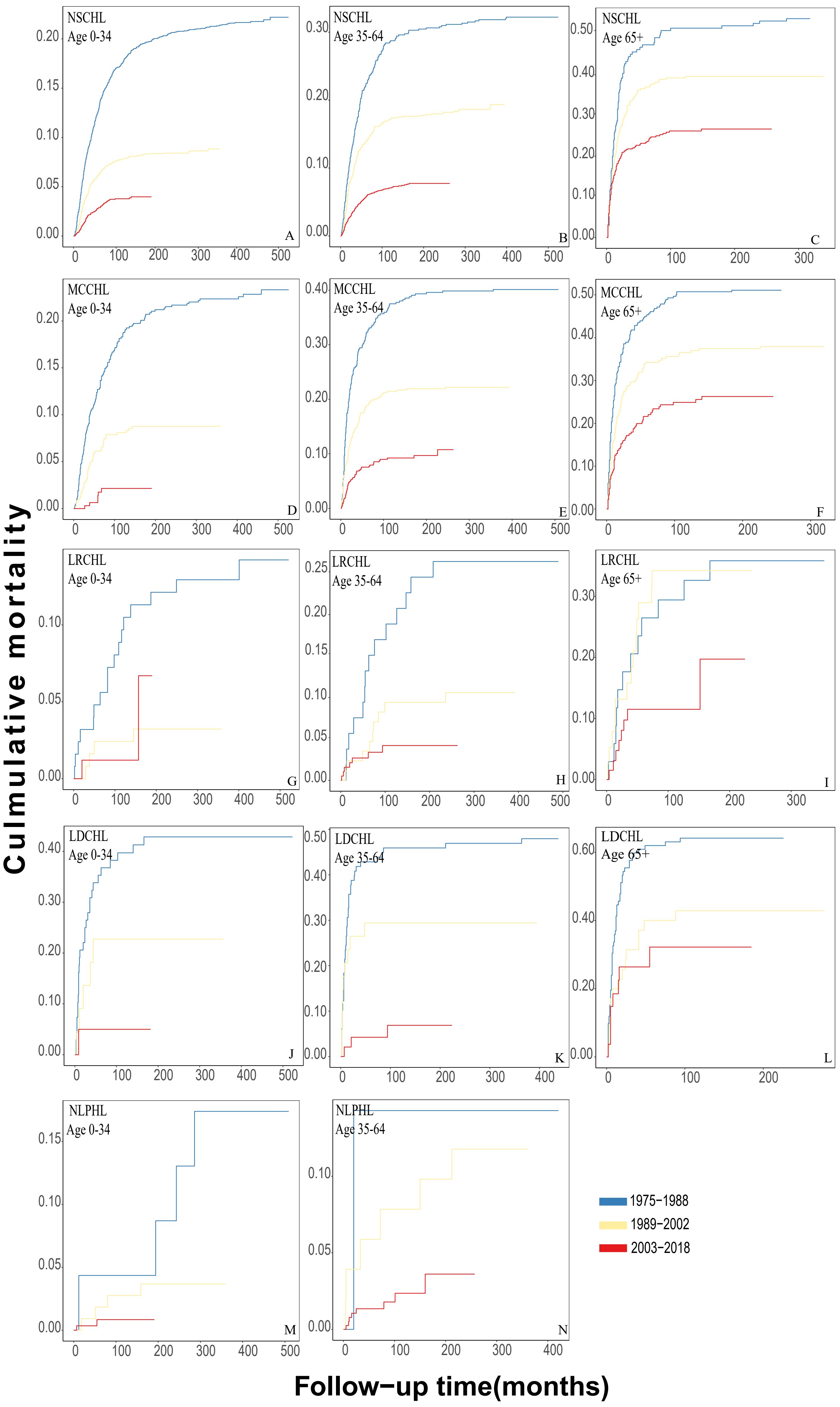

Figure S6. The cumulative HL mortality in different periods. A-N, Patients with different subtypes were further classified according to age. HL Hodgkin lymphoma, CVD cardiovascular disease, cHL classical Hodgkin lymphoma, NSCHL nodular sclerosis cHL, MCCHL mixed-cellularity cHL, LRCHL lymphocyte-rich cHL, LDCHL lymphocyte-depleted cHL, NLPHL nodular lymphocyte-predominant HL.

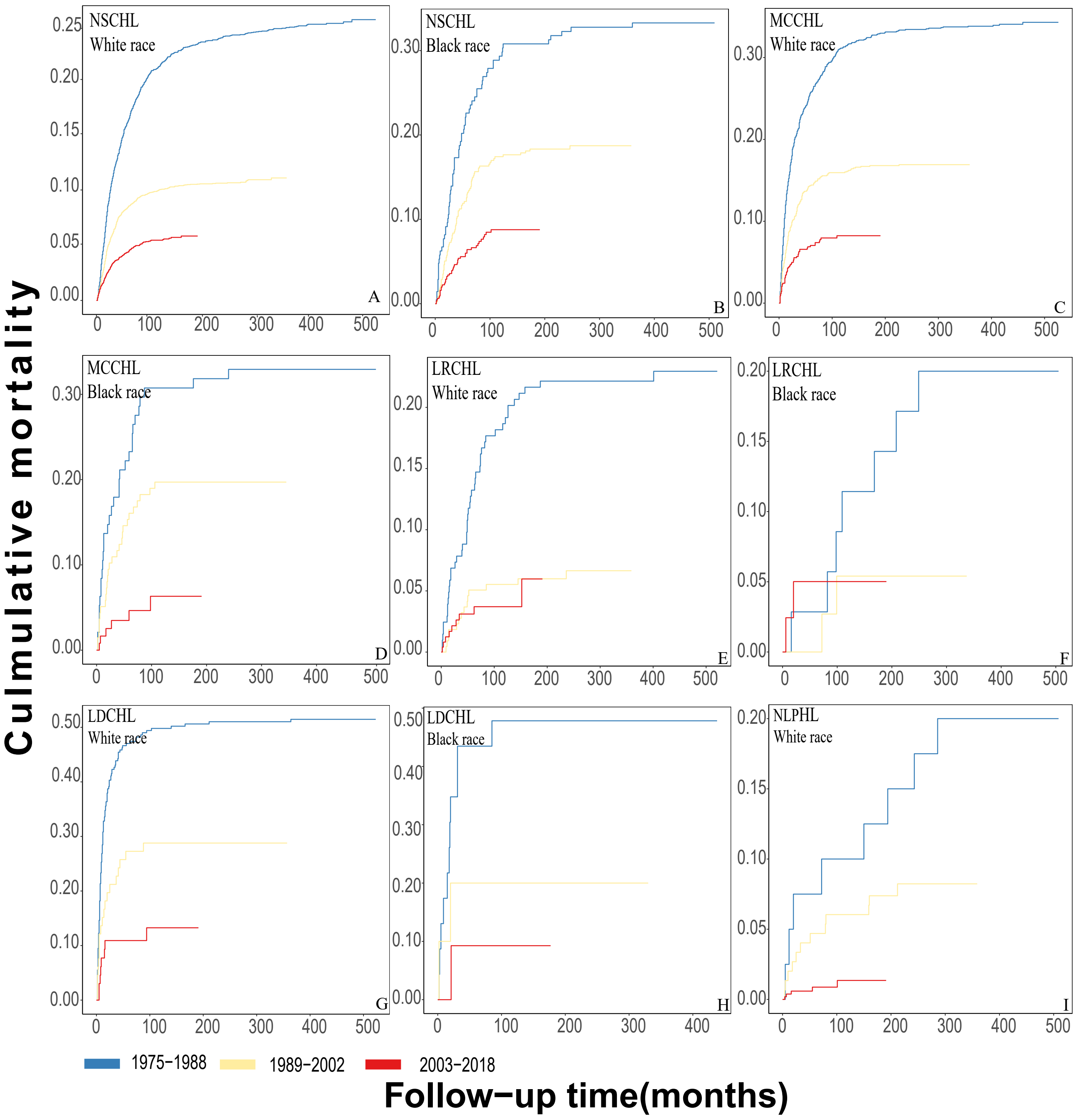

Figure S7. The cumulative HL mortality in different periods. A-I, Patients with different subtypes were further classified according to race. HL Hodgkin lymphoma, CVD cardiovascular disease, cHL classical Hodgkin lymphoma, NSCHL nodular sclerosis cHL, MCCHL mixed-cellularity cHL, LRcHL lymphocyte-rich cHL, LDCHL lymphocyte-depleted cHL, NLPHL nodular lymphocyte-predominant HL.

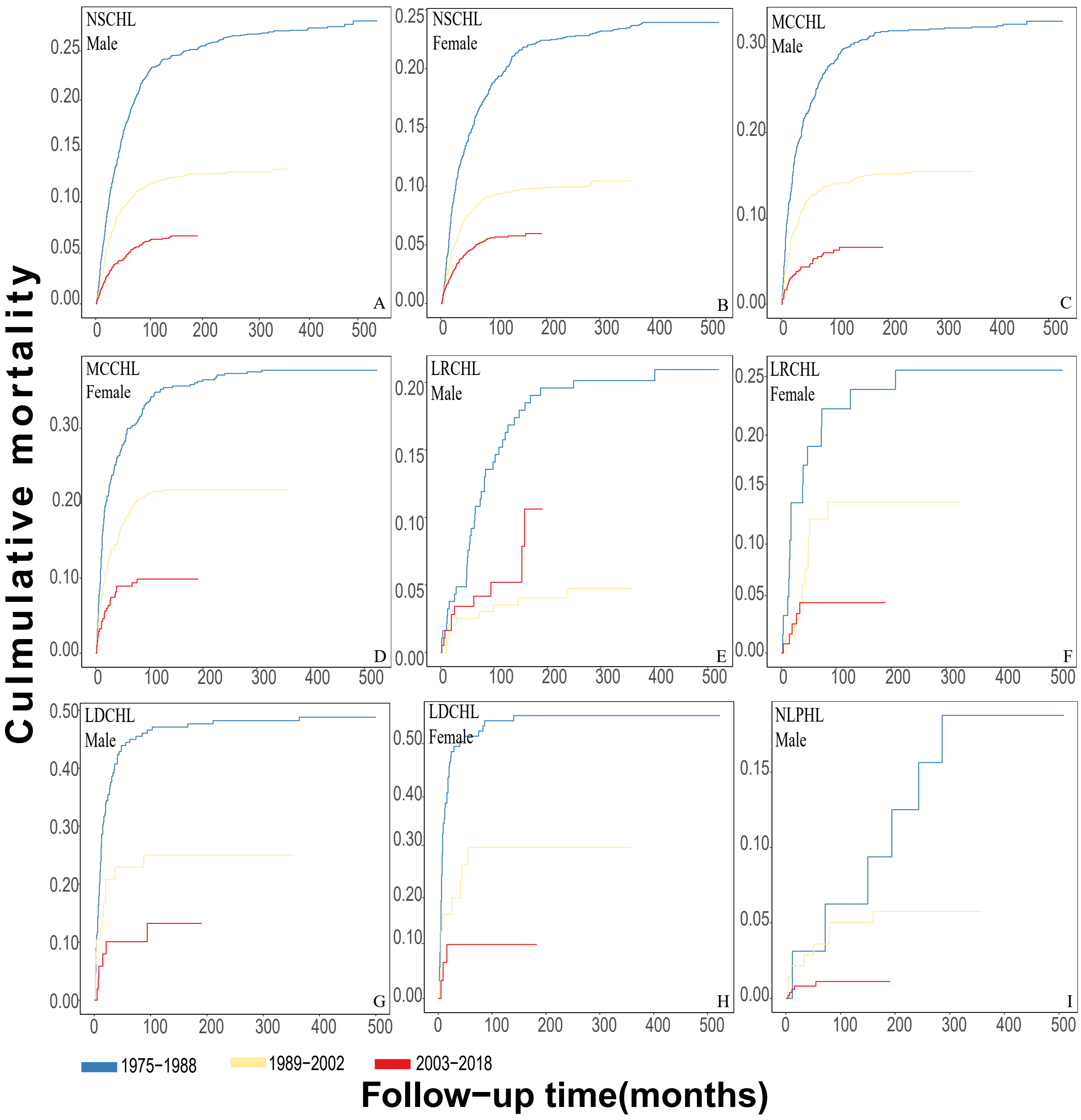

Figure S8. The cumulative HL mortality in different periods. A-I, Patients with different subtypes were further classified according to sex. HL Hodgkin lymphoma, CVD cardiovascular disease, cHL classical Hodgkin lymphoma, NSCHL nodular sclerosis cHL, MCCHL mixed-cellularity cHL, LRCHL lymphocyte-rich cHL, LDCHL lymphocyte-depleted cHL, NLPHL nodular lymphocyte-predominant HL.

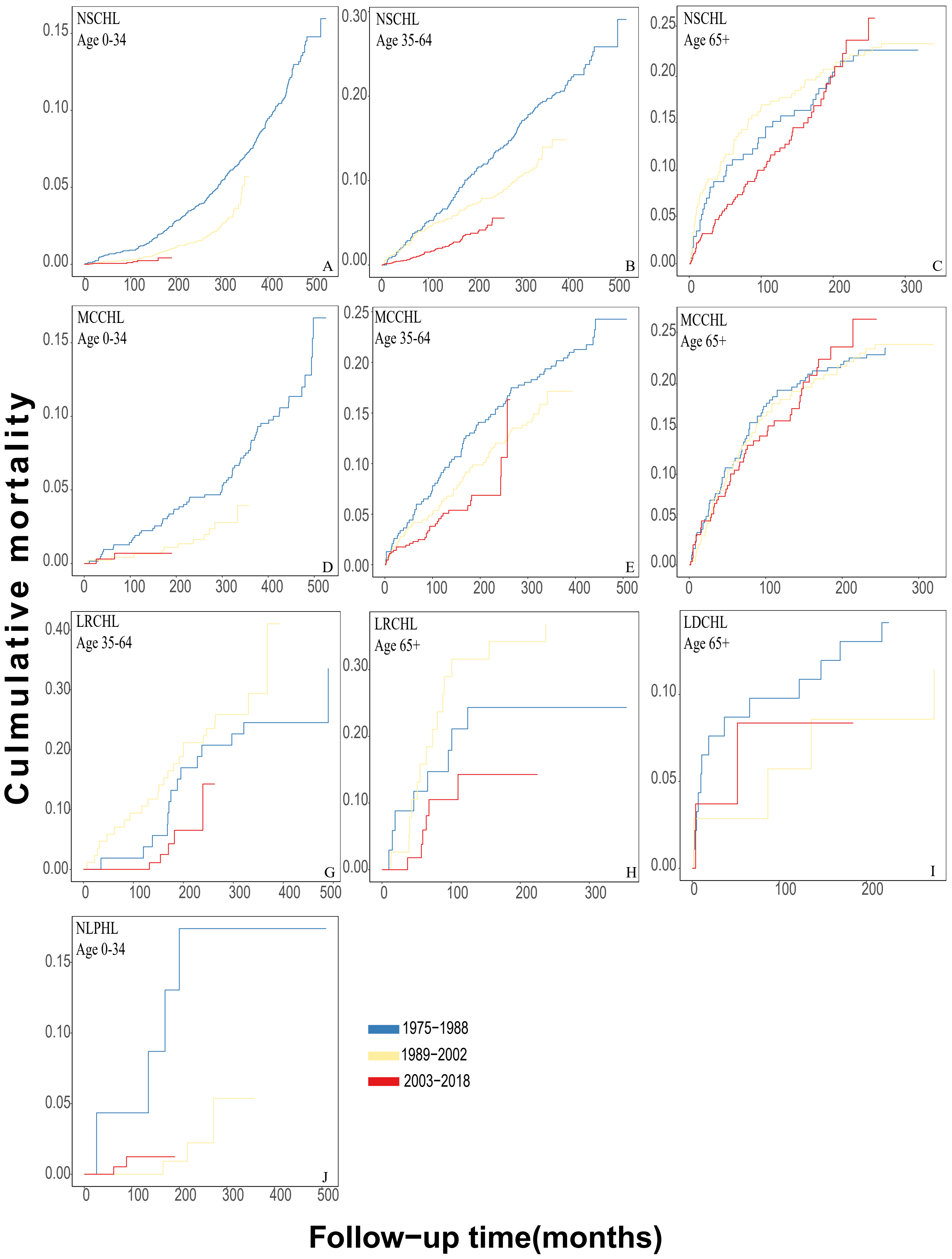

Figure S9. The cumulative CVD mortality in different periods. A-J, Patients with different subtypes were further classified according to age. HL Hodgkin lymphoma, CVD cardiovascular disease, cHL classical Hodgkin lymphoma, NSCHL nodular sclerosis cHL, MCCHL mixed-cellularity cHL, LRCHL lymphocyte-rich cHL, LDCHL lymphocyte-depleted CHL, NLPHL nodular lymphocyte-predominant HL.

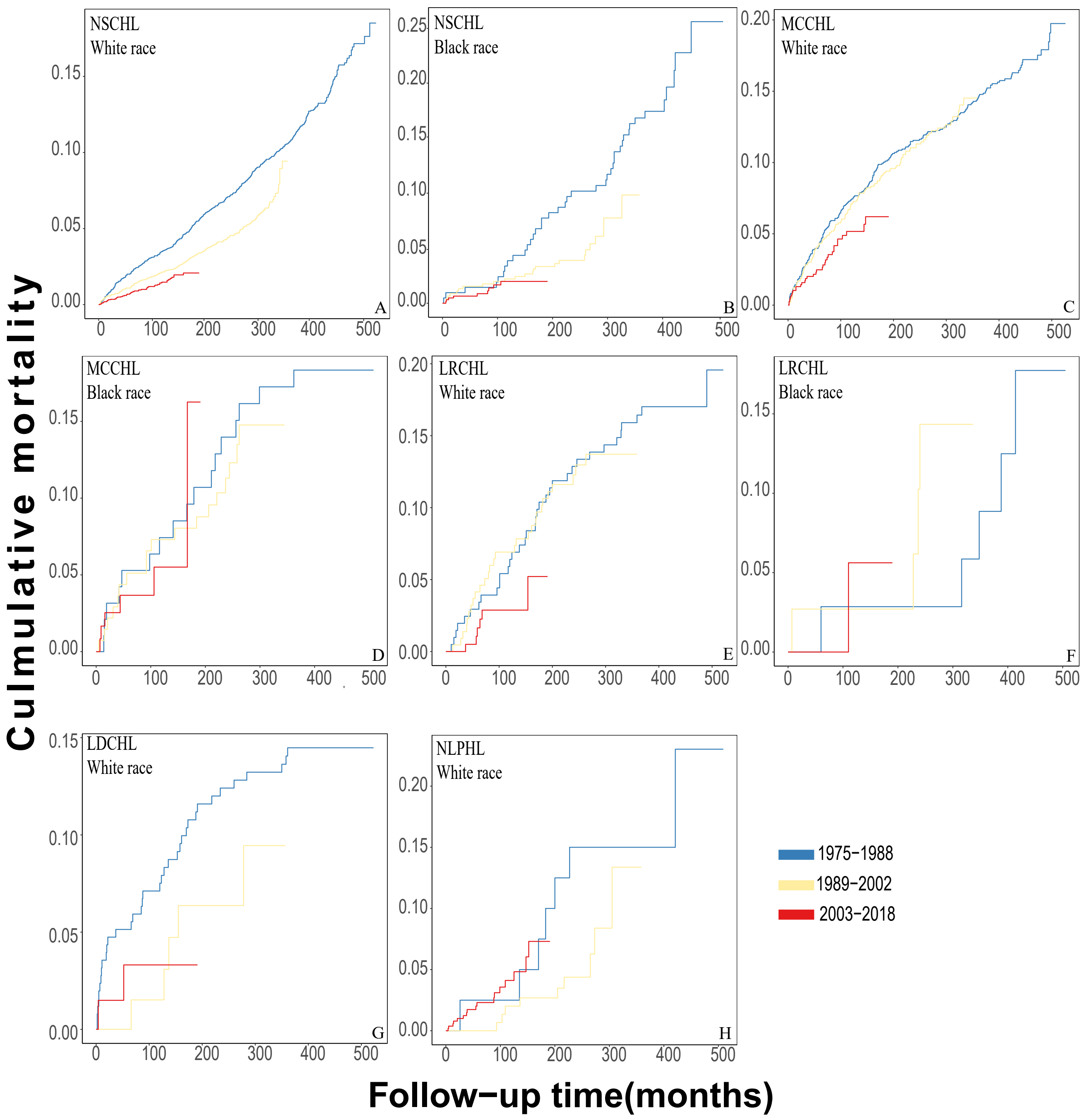

Figure S10. The cumulative CVD mortality in different periods. A-H, Patients with different subtypes were further classified according to race. HL Hodgkin lymphoma, CVD cardiovascular disease, cHL classcial Hodgkin lymphoma, NSCHL nodular sclerosis cHL, MCCHL mixed-cellularity cHL, LRcHL lymphocyte-rich cHL, LDCHL lymphocyte-depleted cHL, NLPHL nodular lymphocyte-predominant HL.

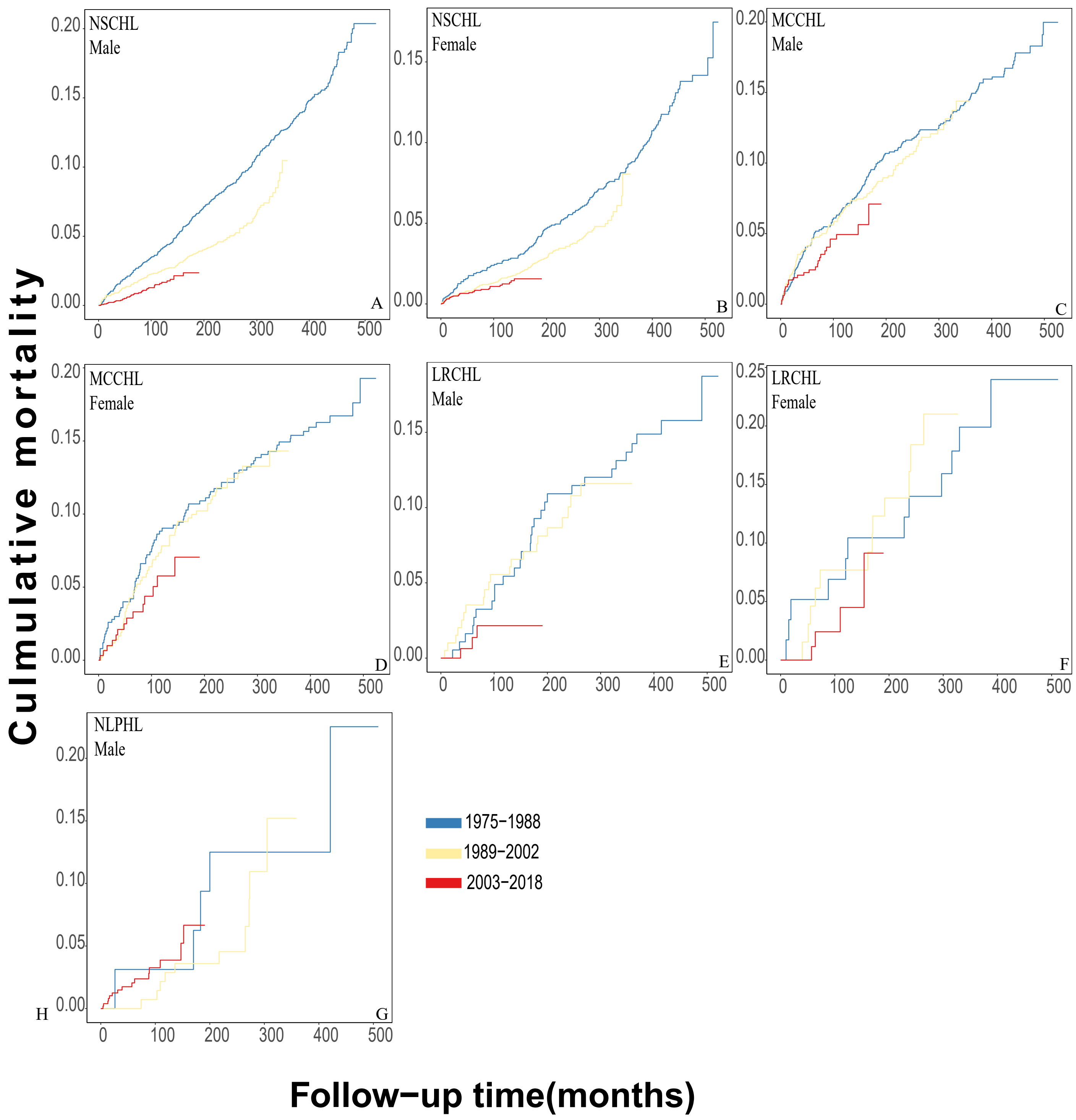

Figure S11. The cumulative incidence of CVD mortality in different periods. A-G, Patients with different subtypes were further classified according to sex. HL Hodgkin lymphoma, CVD cardiovascular disease, cHL classical Hodgkin lymphoma, NSCHL nodular sclerosis cHL, MCCHL mixed-cellularity cHL, LRCHL lymphocyte-rich cHL, LDCHL lymphocyte-depleted cHL, NLPHL nodular lymphocyte-predominant HL.
